# Improving Patient Care and Clinical Services: Compliance With the National Institute for Health and Care Excellence (NICE) Guidelines for Venous Thromboembolism Prophylaxis After Hip and Knee Arthroplasty

**DOI:** 10.7759/cureus.86448

**Published:** 2025-06-20

**Authors:** Raja Muhammad Mussab, Mohanraj Venkatesan, Iftikhar Ahmad Khan, Vishnu Raja, Siddharth Kothari

**Affiliations:** 1 Orthopaedics and Trauma, Jinnah Postgraduate Medical Centre, Karachi, PAK; 2 Orthopaedics and Trauma, Russells Hall Hospital, Dudley, GBR; 3 Orthopaedics, Russells Hall Hospital, Dudley, GBR

**Keywords:** clinical audit, dvt prevention, hip and knee replacement, national institute for health and care excellence (nice), pulmonary embolism (pe), service improvement, total hip replacement (thr), total knee replacement (tkr), vte prophylaxis

## Abstract

Introduction

Venous Thromboembolism (VTE) prophylaxis is an integral part of post-operative management in trauma and orthopedic surgery. Post-operative VTE prophylaxis, especially in lower limb large joint arthroplasty (LLLJA), helps prevent deep vein thrombosis (DVT) and pulmonary embolism (PE), both of which can lead to significant morbidity and mortality. We audited our practice against the National Institute for Health and Care Excellence (NICE) guidelines for prescribing VTE prophylaxis in post-operative patients who had undergone LLLJA at our hospital.

Methods

This was a closed-loop audit evaluating 95 patients, including 49 who underwent total hip replacements (THR) and 46 who had total knee replacements (TKR). In the first cycle, we reviewed discharge summaries for 48 patients (25 THR and 23 TKR) from January 2024. We presented our initial audit results in May 2024 and introduced four corrective measures. Two months later, in July 2024, we re-audited a further 47 patients (24 undergoing THR and 23 undergoing TKR) and applied statistical analysis to evaluate the changes between the two cycles.

Results

During the first cycle in January 2024, 23 (92%) out of the 25 patients who underwent THR and 20 (87%) out of 23 patients who underwent TKR received VTE prophylaxis in accordance with the NICE guidelines (enoxaparin and anti-embolism stockings). Following implementation of the arthroplasty discharge pack, educational sessions, and nurse-led prescription reviews, the second cycle demonstrated notable improvement, with all 24 (100%) of patients who underwent THR and 22 (96%) of the 23 patients who underwent TKR were managed correctly, resulting in an overall rise in compliance from 90% to 98%.

Conclusion

The introduction of the arthroplasty discharge pack, alongside regular teaching for staff, and final checks by the nurse in charge, increased compliance with the NICE guidelines for VTE prophylaxis in elective hip and knee arthroplasty.

## Introduction

A venous thromboembolism (VTE) refers to a condition in which a blood clot impedes venous circulation. It may manifest as deep vein thrombosis (DVT), in which a thrombus lodges within the deep veins of the lower or upper limbs, or as a pulmonary embolism (PE), wherein a part of the clot migrates to the pulmonary arterial system [1]. VTE ranks as the third most prevalent cardiovascular disorder after stroke and myocardial infarction [2], and its incidence rises with advancing age, occurring in roughly one per 10,000 younger individuals compared with one per 100 older adults [2]. 

The pathogenesis of VTE is encapsulated by Virchow’s Triad, encompassing endothelial injury, a hypercoagulable state, and venous stasis [3]. Patients recovering from lower limb large joint arthroplasty (LLLJA) face heightened VTE risk, as surgery contributes to each element of this triad [4]. PE remains the most serious sequela of VTE, although afflicted patients may also experience stroke, syncope, seizures, arrhythmias, heart failure, pulmonary hypertension, post-thrombotic syndrome, and chronic venous insufficiency [5]. 

Administration of VTE prophylaxis significantly reduces thromboembolic events in the post-operative setting. This approach combines pharmacological measures, notably low-molecular-weight heparin (LMWH), with mechanical methods such as anti-embolism stockings (AES) [6]. According to the National Institute for Health and Care Excellence (NICE) guidance, patients undergoing total hip replacement (THR) require a 28-day regimen of LMWH alongside AES until hospital discharge, whereas those receiving total knee replacement (TKR) should continue both interventions for 14 days post-operatively [7]. 

The purpose of this closed-loop audit is to assess the accuracy of VTE prophylaxis being prescribed in accordance with the NICE criteria, to determine institutional compliance and to introduce corrective strategies aimed at optimizing adherence. 

## Materials and methods

This retrospective, observational, closed-loop audit was conducted at Russells Hall Hospital, a District General Hospital in Dudley, UK. It was registered with the Trust Audit Registry under registration number T&O/QI/2024-25/21. The audit standard was the NICE guidelines for VTE prophylaxis in patients undergoing elective hip and knee replacement. 

The first audit cycle was conducted in January 2024, assessing a randomly selected sample of 48 patients (25 THR and 23 TKR). Following the implementation of corrective measures, the second audit cycle took place in July 2024 and included 47 additional patients (24 THR and 23 TKR). Patients with LMWH or AES allergies and those with a hospital stay exceeding five days were excluded. 

Data were collected from electronic discharge summaries available on the hospital computers, and included the operation date, discharge date, operative details, allergies, discharge summaries, specific duration of LMWH, its dose, and AES. 

Data analysis was performed using Microsoft Excel (Microsoft Corp., Redmond, WA). The descriptive statistics presented the VTE prophylaxis compliance in the form of percentages and frequency. In addition, the total compliance rate was calculated with the help of the average compliance rate of TKR and THR. Before the second audit cycle, the initial findings were presented at the local trauma and orthopedic department's monthly audit meeting. Corrective interventions were discussed and implemented in May 2024, including the creation of an arthroplasty discharge pack, placement of these packs as posters and handouts in the arthroplasty station, regular teaching sessions for relevant healthcare workers on NICE discharge guidelines, and final checks of discharge letters by the nurse in charge to identify prescribing errors. 

## Results

During the first cycle (January 2024), 25 (100%) patients underwent THRs. Of these, 23 (92%) received both enoxaparin and AES, as shown in Table 1.

**Table 1:** Distribution of the methods of prophylaxis for patients undergoing THR across Cycles 1 and 2 of the audit AES: Anti-embolism stockings

Prophylaxis	Cycle 1	%	Cycle 2	%
Enoxaparin + AES	23	92.0%	24	100.0%
Enoxaparin only	1	4.0%	0	0.0%
AES only	1	4.0%	0	0.0%
Total	25	100.0%	24	100.0%

One patient received enoxaparin alone, and another received only AES. After the intervention (July 2024), 24 (100%) patients who underwent THR were audited in the second cycle, and all of them received the combined prophylaxis of enoxaparin plus AES, achieving full compliance.

In the TKR cohort, 23 (100%) patients were reviewed during cycle one. A total of 20 (87%) of these patients received both enoxaparin and AES, while two patients received enoxaparin alone, and none received AES alone. One patient did not receive any pharmacological prophylaxis but did have stockings in place. Following the corrective measures, the second cycle again included 23 (100%) patients who had undergone TKR. Of these, 22 (96%) were prescribed the dual regimen, and one patient received enoxaparin only; a single patient received AES alone (Table 2). 

**Table 2:** Distribution of the methods of prophylaxis for patients undergoing TKR across Cycles 1 and 2 of the audit AES: Anti-embolism stockings

Prophylaxis	Cycle 1	%	Cycle 2	%
Enoxaparin + AES	20	87.0%	22	95.7%
Enoxaparin only	2	8.7%	1	4.3%
AES only	0	0.0%	1	4.3%
Total	23	100.0%	23	100.0%

**Figure 1:**
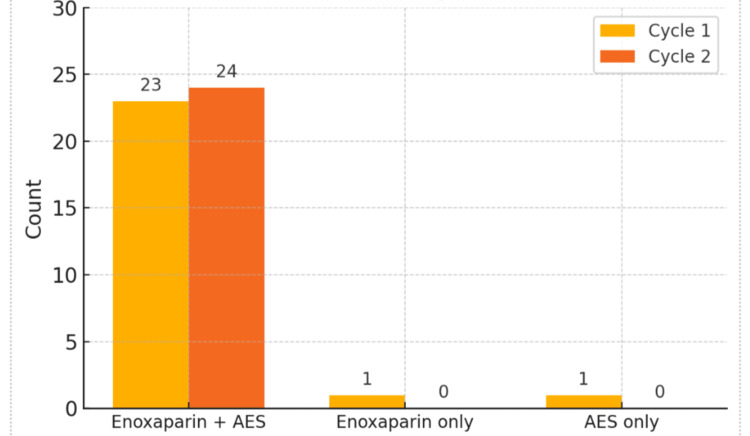
THR count and VTE prophylaxis by cycle THR: Total hip replacement; VTE: Venous thromboembolism; AES: Anti-embolism stockings

**Figure 2:**
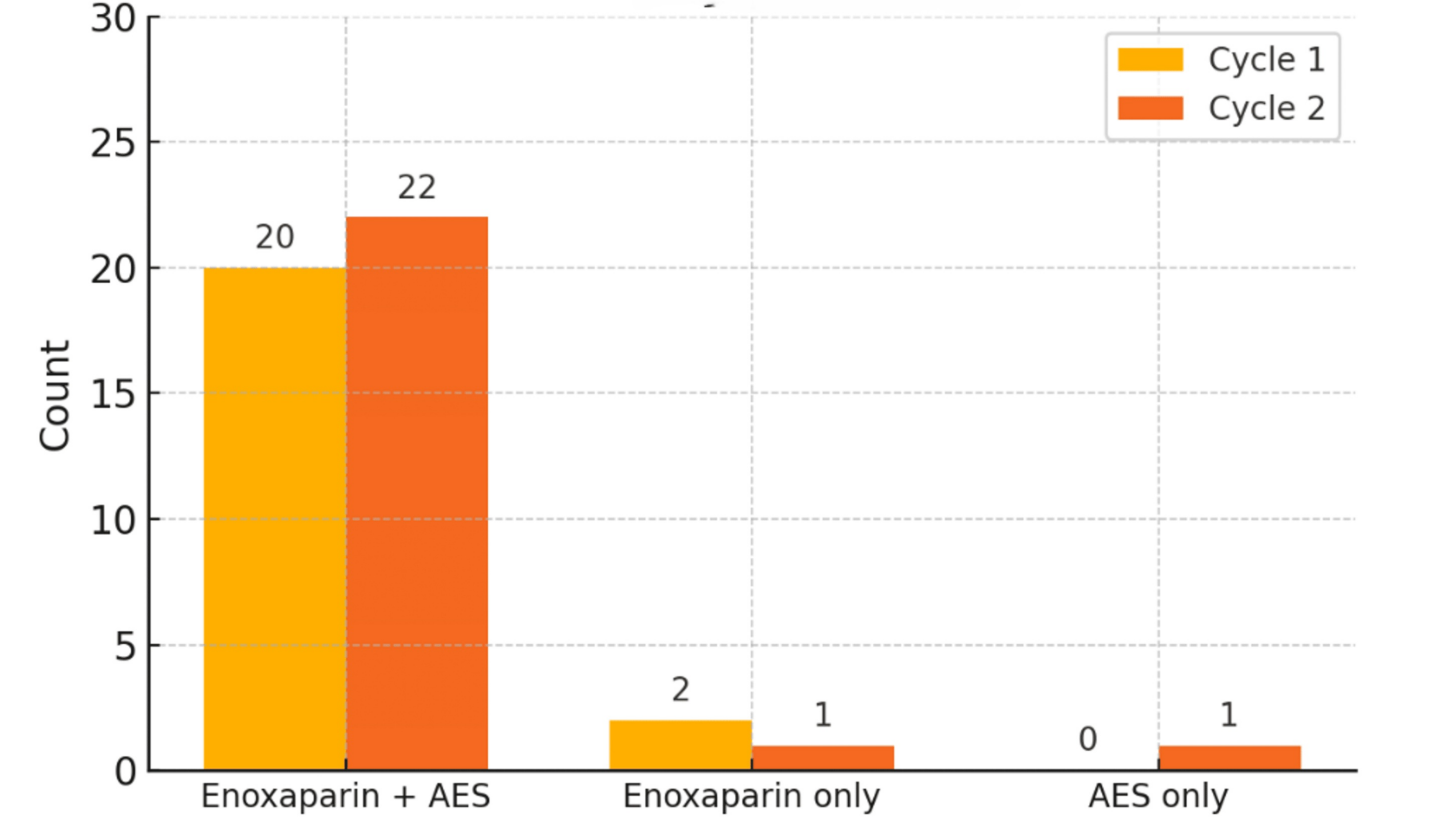
TKR count and VTE prophylaxis by cycle THR: Total knee replacement; VTE: Venous thromboembolism; AES: Anti-embolism stockings

Overall compliance improved between cycles, as shown in Figures 1, 2.

Averaging the THR and TKR cohorts, the compliance rate rose from 90% in the first cycle to 98% in the second. This shift reflects a substantial reduction in prescribing omissions, most notably in achieving 100% adherence among patients undergoing THR and near-perfect adherence among those undergoing TKR. 

## Discussion

Our closed-loop audit showed an improvement in our practices after multiple interventions were implemented. The prescribing of VTE prophylaxis, including LMWH and AES, improved among patients undergoing TKR and THR. Our results were comparable to or better than those found at other centers. A study carried out in the USA estimating the prescribing efficiency for VTE prophylaxis in patients undergoing LLLJA showed 93.8% efficiency in prescribing a pharmacological agent and 55.8% efficiency in prescribing an AES [8]. Other studies show a compliance of 85% even after conducting interventions in their institutes [9]. 

The combined compliance rate of 98% in the second cycle substantially exceeds that reported in several other audits of orthopedic VTE prophylaxis. A single-center review found overall compliance of 77% for pharmacological and mechanical prophylaxis combined, with pharmacological adherence of 72% and mechanical adherence of 81% [10]. An Australian multicenter cohort study observed a wide variation in guideline adherence and a 35% compliance rate for VTE prophylaxis overall, although this figure encompassed both antibiotic and VTE measures [11]. In comparison, our initial compliance of 90% in the first cycle aligns with these benchmarks, but our performance in the second cycle clearly surpasses them. 

Compliance with correct VTE prophylaxis, both by the patient and by the prescriber, has an inverse relation to VTE development in such patients [12]. Furthermore, adequate prescription will also aid in a same-day discharge for such patients and fewer post-operative complications [13]. However, Badge et al. found that failure to follow the guidelines did not reduce the risk of symptomatic VTE within 90 days [14]. It is important to notice that Badge et al. [14] followed the Australian Orthopaedic Association guidelines and not the NICE guidelines, like our study. 

In our case, the improvement noted was likely the result of the creation of an arthroplasty discharge pack and also due to the senior station nurses' counterchecking of the discharge letters for any missed VTE prophylaxis, as also seen in [11]. Nonetheless, a study reported that non-compliant VTE prophylaxis doubled the odds of post-operative VTE and surgical site infection [11]. Moreover, delayed initiation of VTE prophylaxis (beyond 12 hours after major orthopedic surgery) doubled the odds of in-hospital VTE, as identified by Kobzeva-Herzog et al. [15]. Jones et al. also showed evidence that patients with interrupted VTE prophylaxis had a much higher risk of post-operative VTE, though many interruptions were medically justified [16]. 

Our near-perfect adherence suggests a potential reduction in avoidable complications, even though our audit did not capture patient-level outcomes. An audit of lower-limb orthopedic surgery reported pharmacological prophylaxis adherence of 82% and mechanical adherence of 88%, with overall compliance of 85% [17]. This again falls below our rate during the second cycle. The interventions we introduced appear to have been effective. The arthroplasty discharge pack functioned as a cognitive aid at the point of prescription and discharge. This echoes findings from a quality-improvement project in the UK that combined wall posters, checklists, and audit feedback to raise compliance from 40% to 79% over six months [18]. Regular teaching sessions have been shown to enhance knowledge retention and guideline uptake among junior doctors and nursing staff, while senior-led discharge checks provide an additional safety net [10]. Our approach of combining education, visual prompts, and final-check authorization aligns with best-practice recommendations for sustained behavioral change. 

Several factors limit the generalizability of the study findings. The audit’s retrospective design depended on discharge summaries that may have omitted duration or dosage. A single-center approach reflects local resources, culture, and small cohorts risk type II error. We also did not record patient outcomes, seasonal changes, and staff rotation, which might have influenced compliance, independent of the interventions. 

## Conclusions

The audit demonstrated that a structured, multimodal intervention can improve adherence to NICE guidelines for VTE prophylaxis in elective hip and knee arthroplasty. Compliance increased after the introduction of an arthroplasty discharge pack, targeted teaching sessions, and nurse-led prescription checks. These measures effectively eliminated most prescribing omissions, achieving full compliance in patients undergoing THR and near-perfect rates in those undergoing TKR. Although patient outcomes were not captured, the higher adherence rates suggest a reduced risk of preventable thromboembolic complications. Future audits with a larger patient volume, multicenter, and duration-specific should incorporate clinical endpoints and must be expanded to other surgical specialties to confirm and sustain these gains. 
